# Applications of Bolm’s Ligand in Enantioselective Synthesis

**DOI:** 10.3390/molecules25040958

**Published:** 2020-02-20

**Authors:** Eva Bednářová, Štefan Malatinec, Martin Kotora

**Affiliations:** Department of Organic Chemistry, Faculty of Science, Charles University, Albertov 6, 128 43 Praha 2, Czech Republic

**Keywords:** catalysis, enantioselectivity, synthesis, bipyridine

## Abstract

One pathway for the preparation of enantiomerically pure compounds from prochiral substrates is the use of metal complex catalysis with chiral ligands. Compared to the other frequently used chiral ligands, chiral 2,2′-bipyridines have been underexploited, despite the data indicating that such ligands have considerable potential in synthetic chemistry. One of those is the so-called Bolm’s ligand, a compound possessing chiral alcohol moieties in the side chains attached to the 2,2′-bipyridine scaffold. Various metal salts have been used in combination with Bolm’s ligand as potent catalysts able to bring about enantioselective alkylations, allylations, conjugate additions, desymmetrization of *meso*-epoxides, aldol reactions, etc. This review aims to summarize Bolm’s ligand applications in the area of enantioselective synthesis over the last three decades since its preparation.

## 1. Introduction

Growing demands for chiral compounds in pharmaceutical, agrochemical, and other fields of chemical industry have resulted in the search for new, more selective, economical, and environmentally friendly approaches in asymmetric synthesis. In this respect, enantioselective catalysis by means of chiral metal complexes is one such approach. Endeavors in this field are closely associated with the development of new chiral ligands that are able to modulate the reactivity of the metal center in such a way that the prochiral compound is selectively transformed into one out of two possible enantiomers. In comparison to other classes of chiral ligands based on phosphines, salens, oxazolines, or the tartrate scaffolds, chiral 2,2′-bipyridines have attracted comparatively little attention.

Their low popularity probably stems from the lack of suitable synthetic methods furnishing substituted chiral 2,2′-bipyridines. Fine-tuning of such bipyridines by judicious substitution could improve the activity of the whole metal-ligand complex catalyst and thus provide chiral products of catalyzed reactions in higher yields and enantiopurities. The development of methods providing such substituted bipyridines is thus desirable.

In general, 2,2′-bipyridines have found application in various fields of chemistry—such as macromolecular chemistry [1], supramolecular chemistry [2], material chemistry [3], photochemistry [4], electrochemistry [5]—and, due to their extraordinary coordination properties, as ligands in metal-catalyzed reactions [6]. Compared to other commonly used ligands, such as phosphines and cyclopentadienes, they are remarkably stable towards both air and moisture, which is advantageous during their preparation, handling, and storage. The presence of two nitrogen atoms in an almost ideal configuration to form the five-membered chelate ring with a metal center results in remarkably stable complexes even with labile metal sources. In addition, the derivatization of one of the other eight carbon atoms, thanks to the change of steric and electronic properties, enables the achievement of the optimal behavior of the whole complex for the appropriate application [7]. 

**Figure 1:**
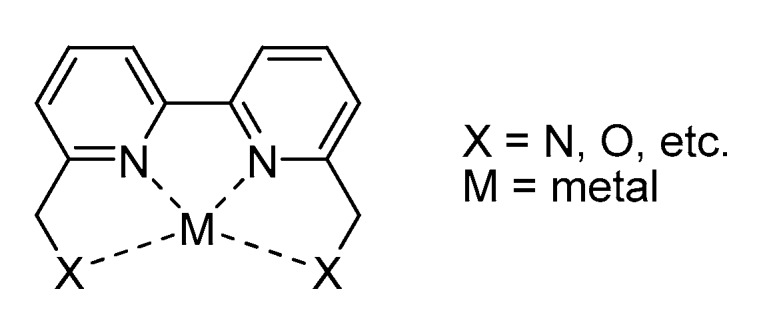
Multidentate chelation of metal center by 6,6′-substituted 2,2′-bipyridine.

The substitution of the 2,2′-bipyridine scaffold in the positions 6 and 6′ causes the most significant changes in the coordination abilities of the corresponding bipyridine. The bulky substituents increase steric hindrance, which hampers the accessibility of the two nitrogen atoms towards coordination. On the other hand, a finely tuned substituent with atoms capable of chelation of the metal center can stabilize the whole complex through the creation of multicoordinated complexes (Figure 1). Moreover, the insertion of a side chain bearing the center of chirality allows for the control of the stereochemistry around the metal center and thus expands the application scope to asymmetric catalysis. During the last decades, chiral 2,2′-bipyridines have found application in the cyclopropanation of alkenes, alkylation and allylation of aldehydes, hydrogenation and hydrosilylation of carbonyl compounds, alkylation and oxidation of allylic position, aldol type reactions, etc. [8].

Substituted 2,2′-bipyridines can be divided into two groups: those with C_2_- and C_1_-symmetry elements. Examples of bipyridines possessing a central, axial, planar, and helical element of chirality can be found, as well as those with more than one element of chirality. However, the most significant are 2,2′-bipyridines with C_2_-symmetry element.

## 2. Bolm’s Ligand

### 2.1. Bolm’s Ligand Properties and Synthesis

**Figure 2:**
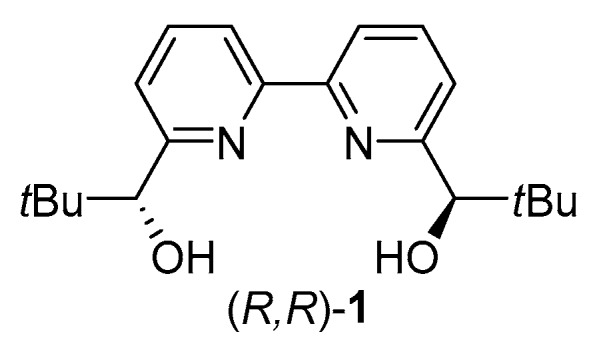
Bolm’s ligand (*R,R*)-**1**.

The most frequently used chiral 2,2′-bipyridine ligand, and perhaps the most versatile one used in metal-catalyzed asymmetric reactions, is Bolm’s ligand **1**. It has C_2_-symmetry and possesses the bipyridine scaffold with two side chains with chiral alcohols moieties in positions 6 and 6′ (Figure 2). Due to its ability to chelate several metallic ions, Bolm’s ligand **1** has found application in various types of metal-catalyzed enantioselective reactions.

(*R,R*)-**1** was designed and synthesized by Bolm et al. in 1990 [9], and a more detailed synthesis was then described in 1992 by the same authors [10]. The synthesis started from commercially available 2,6-dibromopyridine and was accomplished in four steps. Later, the synthesis was improved by Kobayashi et al., who developed an alternative method for its preparation [11]. 

NMR studies of Bolm’s ligand **1** in a solution, as well as a single crystal X-ray diffraction analysis, confirmed the presence of the *transoid* arrangement of nitrogen atoms in pyridine rings. Nevertheless, the nitrogen atoms can also adopt, due to the free rotation around the C2–C2′ bond, a *cisoid* arrangement, which is actually the favorable conformation in its complexes with metal salts. Usually, both nitrogen atoms are coordinated to the same metal center [10]. 

The presence of two nitrogen atoms in the pyridine rings and two oxygen atoms in the side chains indicates that Bolm’s ligand could theoretically form mono-, bi-, tri- or tetracoordinated metal complexes. While mono- and bicoordinated complexes of Bolm’s ligand are not known, the formation of the respective tri- and tetracoordinated complexes have been observed. The tricoordinated complexes of **1** are formed with Zn(II) bromide, as well as with Cu(II) bromide, and the complexes adopt a square-pyramidal arrangement where nitrogen and oxygen atoms of **1** are attached to the same metal center [12,13]. On the other hand, the Fe(II)- [14,15,16], Sc(III)- [17], Bi(III)- [18], and In(III)-complexes [19] have a pentagonal bipyramidal arrangement, in which both nitrogen atoms of the bipyridine scaffold and both oxygen atoms of the hydroxy groups are coordinated in the metal center in the tetracoordinated manner. The four atoms of **1** (N,N,O,O) occupy four equatorial sites of the metal center. This arrangement was confirmed by a single crystal X-ray diffraction analysis. In a similar manner, Y(III) also forms a tetracoordinated complex. However, the complex adopts a dodecahedral conformation, because of the eight-coordination fashion of Y(III) cation. It should be noted that both of the hydroxyl protons are retained in all examples of the metal complexes and that the resting coordination sites are occupied by either anions of the metal salts or molecules of solvents.

### 2.2. Alkylation and Allylation of Aldehydes

**Scheme 1:**
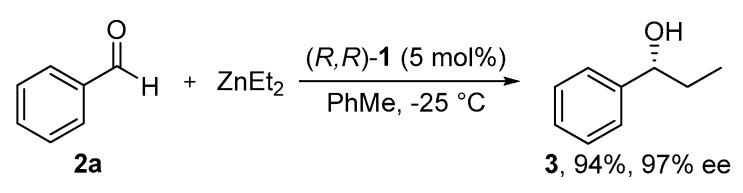
Alkylation of benzaldehyde **2a**.

In its first application, Bolm’s ligand was used in the enantioselective alkylation of aldehydes using diethylzinc as the alkylating agent [9]. With 5 mol% of (*R,R*)-**1** in toluene at −25 °C, the ethylation of benzaldehyde **2a** yielded the secondary alcohol **3** in 94% with 97% ee (Scheme 1). In the following work, the scope of the reaction was assessed by using a wide range of aldehydes under similar reaction conditions [20]. While the aromatic aldehydes provided appropriate products in high yields and enantiomeric purities (79–97% ee), the aliphatic aldehydes were alkylated in lower yields and enantioselectivities (16–70% ee).

The first examples of allylation of aldehydes were described by Kobayashi et al. in 2010 and 2011 [21,22]. Whereas the uncatalyzed allylation led predominantly to the γ-addition product **7** (Entry 1, Table 1), the one catalyzed by Zn(II) hydroxide shifted the regioselectivity towards α-product **6** (Entry 2). The exclusive formation of α-addition products **6** was observed when the reaction was catalyzed by a combination of Zn(II) hydroxide and Bolm’s ligand (*S,S*)-**1** (Entry 3). In addition, the *syn*-product was obtained predominantly with 71% ee.

The assessment of the scope of the reaction revealed that other aldehydes are also allylated, forming exclusively α-addition products (Table 2). 3-Phenylpropanal **2b** was α-(alkyl)allylated using various boronates **5a-5c** in excellent yields and high to excellent enantioselectivity (Entries 1–3), as well as α-chloroallylation with **5d** proceeded smoothly with 3-phenylpropanal **2b** (Entry 4), benzaldehyde **2a** (Entry 5), substituted benzaldehydes **2c** and **2d** (Entries 6 and 7), 1-naphthylcarbaldehyde **2e** (Entry 8), and undecanal **2f** (Entry 9). In all cases, α-chlorohomoallylic alcohols were obtained with a selectivity higher than 93% of the *syn* product.

The mechanism of the described allylation has not yet been completely elucidated, but the authors proposed a double γ-addition process where the boron atom is in the first step transmetallated by zinc in γ-addition fashion, while the formed allylzinc species reacts in the next step with aldehyde again by γ-addition. Some of the intermediates of the proposed mechanism were confirmed by mass spectrometry [12]. 

### 2.3. Conjugate Addition

**Scheme 2:**
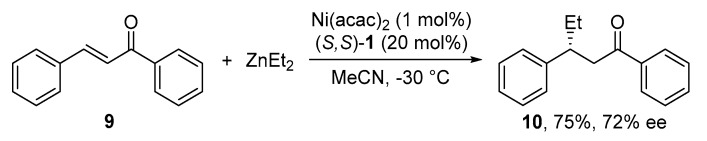
Conjugate addition to enone **9**.

Conjugate addition is one of the most frequently studied reactions with application of Bolm’s ligand. The first reports were published by Bolm et al. in 1990 and 1992, respectively, and dealt with the ethylation of chalcone **9** with diethyl zinc. The ethylated product **10** was furnished in 75% yield with 72% ee, with only 1 mol% of Ni(II) acetylacetonate, but 20 mol% of ligand (*S,S*)-**1** were required (Scheme 2) [23,24]. 

In 2006 Kobayashi et al. published an example of Michael addition catalyzed by Sc-complex prepared from Sc(III) triflate and ligand (*S,S*)-**1 [25]**. The study of the reaction of β-ketoester **11** with methyl vinyl ketone (MVK) **12** showed that the course of the reaction depended not only on the solvent used (Entries 3–6, Table 3), but also on concentration of the starting material: the lower the concentration, the higher the enantioselectivity (Entries 6–8). Regarding temperature, surprisingly, the optimal turned out to be 40 °C, while at lower temperatures both yield and enantiopurity dropped (Entries 1–3 and 8).

In 2011 two groups, Vaccaro et al. and Kobayashi et al., published independently the addition of benzyl thiol to α,β-unsaturated ketone **14** catalyzed by Sc(III) triflate and Bolm’s ligand **1** in water (Table 4) [26,27]. Both groups noticed that, under ambient conditions, the pH of the reaction mixture was acidic, and decided therefore to carry out the reaction in the presence of a catalytic amount of base. The former used NaOH (Entry 2) and the latter pyridine (Entry 3). In both instances, adducts **15** in comparable yields and enantioselectivities were obtained.

The generality of the sulfa-Michael addition was demonstrated through the screening of various types of substrates, such as aromatic and aliphatic enones and thiols. In most cases, the reaction gave products in high to excellent yields (63–100%) and with high enantioselectivity (76–97% ee). The only exceptions were the additions of thiophenol that proceeded with mediocre enantioselectivity in the range of 44–52% ee. Presumably, the drop was caused by its higher acidity.

**Scheme 3:**
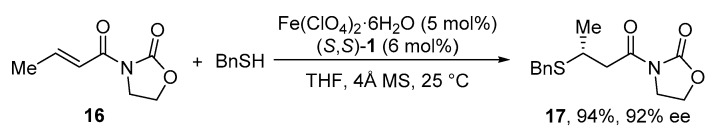
Sulfa-Michael addition of benzylthiol on α,β-unsaturated oxazolidin-2-one **16**.

The sulfa-Michael addition of benzyl thiol to α,β-unsaturated oxazolidin-2-ones catalyzed by a Fe(II)-(*S,S*)-**1** complex has been recently studied by Ollevier et al. They used hexahydrate Fe(II) perchlorate as the Lewis acid [28]. The product **17** of the addition of benzyl thiol on oxazolidin-2-one **16** was isolated in 94% yield with 92% ee (Scheme 3). 

Another type of reaction intensively studied in Kobayashi’s group was the 1,4-addition reaction of bis(pinacolato)diboron (B_2_(pin)_2_) catalyzed by either Cu(II) salts or Cu powder. The addition proceeded on various substrates such as α,β-unsaturated ketones [29,30,31], imines [32], or nitriles [33]. The representative examples of this reaction are summarized in Table 5. The borylated products **18–20** were directly converted to the corresponding β-hydroxy alcohol **21–23**, because of better enantiomer separation and better determination of enantiomeric excess by HPLC analysis. 

Interesting results were obtained with α,β,γ,δ-unsaturated dienone **24** (Table 6) [34]. The 1,6-addition product **26** was obtained by using Cu(II) hydroxide and (*S,S*)-**1** without any additive (Entry 1), but upon using Cu(II) hydroxide and (*S,S*)-**1** with a catalytic amount of acetic acid or copper(II) acetate and (*S,S*)-**1,** 1,4-addition products **25** with negligible effect on enantioselectivity (Entries 2 and 3) were obtained. The authors proposed to explain these phenomena by the difference in the reaction mechanism. The plausible reaction intermediate in reactions using Cu(II) acetate (used as such or formed from Cu(II) hydroxide and acetic acid) is (pin)B–CuOAc, which is at the same time an activating and reacting agent enabling smooth 1,4-addition. In the Cu(II) hydroxide catalyzed reaction, the first copper species activates the carbonyl group, while the second copper-boron species attacks **24** via 1,6-addition. In this case, the steric hindrance of activating species is probably sterically preventing the attack of the position 4.

### 2.4. Opening of Meso-Epoxides

The catalytic enantioselective opening of *meso*-epoxides provides a powerful strategy for the formation of enantiomerically enriched products, with two contiguous chiral centers in one step from readily available achiral compounds. Since the reaction can be catalyzed by Lewis acids, the applicability of metal-**1** complexes in this reaction has been intensively studied by several groups in the last two decades.

The desymmetrization of *meso*-epoxides by amines generally suffers from the non-compatibility of Lewis basic amine and Lewis acidic catalyst, because of their tendency to irreversibly coordinate to one another. Nevertheless, the aminolysis of the epoxide ring provides access to chiral β-amino alcohols, which can be found in many biologically active compounds and chiral auxiliaries. Hence, the enantioselective variant of this reaction is of considerable synthetic interest [35]. 

Kobayashi and Schneider et al. reported that a combination of Bolm’s ligand **1** with Sc(III) triflate in DCM (Entries 1 and 3) [13,36,37], and Sc(III) dodecyl sulfate (DS) (Entry 2) [38], or undecyl sulfonate (UDST, Entry 4) [13] as “Lewis acid surfactant catalysts” (LASC) in water, exhibited high catalytic activity and enantioselectivity in the opening of *meso*-stilbene oxide **29** with aniline **30** (Table 7). In all cases, β-amino alcohol **35** in high to excellent yields and enantioselectivities was isolated. Interestingly, the same reaction catalyzed in the presence of copper or zinc salts provided the products with opposite configuration (Entries 5–8). The observed inversion may be caused by a different conformation of the appropriate metal complexes formed upon chelation with ligand **1**. While Cu(II) and Zn(II) form tricoordinated complexes, which adopt the square-pyramidal structure, Sc(III) is chelated by ligand **1** in tetracoordinated manner with the pentagonal bipyramidal conformation [13]. Vaccaro et al. showed that the reaction can also be catalyzed by LASC generated in situ from Zn(II) triflate [39] or Zr(IV) chloride [40] and sodium dodecyl sulfate (Entries 9 and 10). Last but not least, the In(III) [41] and Fe(II) [16] complexes also appeared to be active Lewis acid catalysts for this reaction (Entries 11 and 12).

Schneider et al. and Kobayashi et al. also demonstrated the applicability of alcohols **31** and **32** (Entries 13 and 14) [36,42,43], thiols **33** (Entries 15–17) [19,43,44], and selenols **34** (Entry 18) [45] in the metal-catalyzed (Sc(III) triflate and In(III) bromide) opening of the epoxide ring. The desired β-hydroxy ethers **36**, sulfides **37**, and selenides **38** were obtained in moderate to high yields and high to excellent enantioselectivities. It should be noted that, because of the undesired deselenylation, the selenolyses of *meso*-epoxides had to be carried out in the dark and with degassed solvents.

The study of the nonlinear effect between the enantiopurity of ligand **1** and the enantiomeric excess of a product was examined in order to reveal details about the catalyst structure in the course of the reaction [13]. While the linear effect was observed in the case of a copper catalyst in DCM or in water, as well as in the case of a scandium catalyst in water, the nonlinear effect was noticed when using a scandium catalyst in DCM [46]. Interestingly, the system again evinced the linear relation when the independently preprepared complexes Sc(III)-(*S,S*)-**1** and Sc(III)-(*R,R*)-**1** were mixed in DCM with only a small excess of one of them in order to make a catalyst with low ee. The authors are assuming from these experiments that while some aggregates are formed during the mixing of Bolm’s ligand **1** and Sc(III) triflate in solution, the Sc-**1** complex is bound irreversibly without any possible dissociation. Despite the indisputable importance of the aminolysis of epoxides, this process under the above mentioned conditions is strictly limited to anilines.

**Scheme 4:**
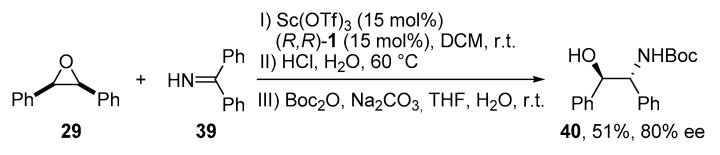
Opening of epoxide **29** with benzophenone imine **39**.

In order to obtain the primary amino alcohols as products of opening of *meso*-epoxides with anilines, one would need to deprotect the *N*-aryl group, which could be problematic. To overcome this, Schneider et al. developed a new approach to obtain these compounds based on the epoxide ring opening by imine **39 [47]**. The products of this reaction were in the next step hydrolyzed and, because of the difficulties in the separation of highly polar 1,2-amino alcohols, protected by the Boc group (*tert*-butyloxycarbonyl). The product **40** was isolated in 51% yield with 80% ee after these three steps using benzophenone imine **39** (Scheme 4). 

A great deal of attention was also paid to the opening of the epoxide ring with indole **41**, because the indole scaffold can be found in many natural products and biologically active compounds [48]. While the reaction utilizing triflates of either Sc(III) (Entry 1, Table 8) or Zn(II) (Entry 6) in DCM provided only traces of the desired product **42**, the reaction with Cu(II) triflate yielded **43** in 60% yield with 86% ee (Entry 4). These results were even improved by using LASC based on Sc(III) (Entries 2 and 3), Cu(II) (Entry 5), or Zn(II) (Entry 7). Again, the inversion of the absolute stereochemistry of the product formed in scandium vs. copper or zinc-catalyzed reactions was observed. The best results in terms of yield and enantioselectivity were obtained with Fe(II) perchlorate (Entry 8), where the indole derivative **42** was obtained in 90% yield and >99% ee.

### 2.5. Mukaiyama Aldol Reaction

Classical aldol reactions may suffer from several possible side reactions such as self-condensation, isomerization, or over-reaction. In this respect, such problems are to a large extent solved by using Mukaiyama aldol reaction conditions. The advantage stems from the fact that the above-mentioned problems are overcome by using stable enols (silyl enol ethers), and the activation of the carbonyl group is generally done by a Lewis acid. In addition, the use of chiral Lewis acids by a combination of a suitable metal cation and a chiral ligand allows the reaction to proceed enantioselectively.

**Scheme 5:**
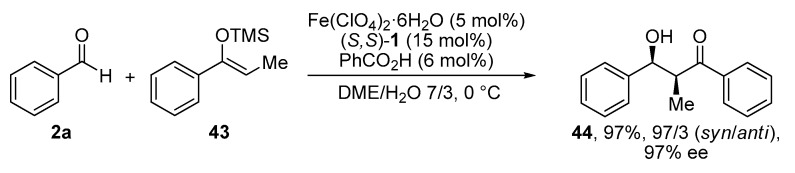
Mukaiyama aldol reaction of **43** with **2a** catalyzed by Fe(II)-(*S,S*)-**1** complex.

Ollevier et al. reported in 2012 a highly enantioselective reaction of **2a** with **43** by using 5 mol% of hexahydrate Fe(II) perchlorate, 15 mol% of ligand (*S,S*)-**1,** and 6 mol% of benzoic acid in a dimethoxyethane (DME)/water mixture (Scheme 5) [14]. Water was found to be essential for the reaction. In its absence, when the reaction was carried out in dry DME, the desired product was not formed. It was shown that these conditions are suitable for various types of substrates including aromatic, aliphatic, heteroaromatic, and unsaturated aldehydes providing products in high yields, diastereoselectivities, and enantioselectivities (exceeding 91% ee for all substrates).

On the occasion of the 40th anniversary of the discovery of the Mukaiyama aldol reaction, Ollevier and Kobayashi et al. published a detailed study on the utilization of Bolm’s ligand **1** in this reaction [49]. They optimized the type of Lewis acid, additive, solvent, ligand, and their loading. They defined three typical conditions (Table 9): conditions A use Fe(II) triflate and pyridine, conditions B Fe(II) perchlorate and benzoic acid, while conditions C are based on Bi(III) triflate and pyridine. Conditions A are suitable for bulky electron-rich aldehydes. On the other hand, bulky electron-poor aldehydes in combination with silyl enol ethers with neutral or electron-withdrawing groups (EWG) are appropriate substrates for conditions B. The bulky, electron-poor aldehydes react well with silyl enol ethers with electron-donating group (EDG) under conditions C, and are also suitable for reaction with non-bulky aldehydes.

**Scheme 6:**
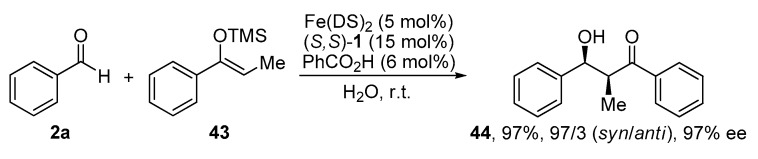
Mukaiyama aldol reaction using LASC conditions.

The feasibility of the Mukaiyama aldol reaction catalyzed by the Fe(II)-(*S,S*)-**1** complex was further improved by Ollevier et al. in 2014 by using LASC (Fe(II) dodecylsulfate) (Scheme 6) [50]. The product of the reaction was isolated in 80% yield with 91/9 dr and 92% ee.

Morokuma et al. performed density functional theory (DFT) and artificial force-induced reaction (AFIR) studies on the system based on Fe(II) triflate and (S,S)-**1**, starting with benzaldehyde **2a** and silyl enol ether **43**, because the Mukaiyama aldol reaction mechanism is a matter of controversy [51]. First, the calculation indicated that a high-spin quintet state of Fe(II)-complex is thermodynamically stable, therefore operating in the whole mechanism. Second, the overall mechanism consisted of six steps. The free energy profile of this reaction revealed that, while the formation of a carbon–carbon bond is the selectivity-determining step, the rate-determining step of the reaction is the proton transfer from the molecule of water to the carbonyl group. Unfortunately, the role of the additive in this study is fully omitted.

### 2.6. Hydroxymethylation

Hydroxymethylation is a very useful transformation, allowing for the introduction of a one-carbon functional group in the α-position of the carbonyl group by using formaldehyde. Since its reaction mechanism is close to that of the aldol reaction, similar catalytic systems could be used to promote it. Kobayashi et al. reported three studies dealing with a Lewis acid catalyzed hydroxymethylation utilizing Bolm’s ligand (*S,S*)-**1** (Table 10). In 2004, the catalysis by Sc(III) triflate in DME/water mixture at −20 °C (Entry 1) was reported [52], and in the following year a Bi(III) triflate catalyzed process at 0 °C was disclosed [18]. In addition, a decrease of the amount of catalyst to only 1 mol% was achieved in the presence of 2,2′-bipyridine as an additive. It is assumed that its presence led to the stabilization of the Lewis acid catalytic species in the aqueous media (Entry 2). The same authors also developed conditions to execute the reaction in water. The presence of Triton^®^ X-705 surfactant was responsible for the formation of a colloidal system (Entry 3) [53]. Under all conditions, a high stereoselectivity of ~90% ee was observed.

### 2.7. α-Amination of β-Ketocarbonyl Compounds

The synthesis of non-natural α-amino acids attracts the attention of a lot of scientific groups because of their potential application in the pharmaceutical industry [54,55]. One of the possible ways for their preparation is the α-amination of carbonyl compounds.

**Scheme 7:**
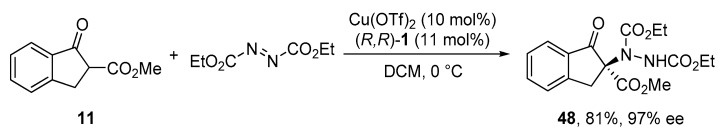
α-Amination of β-ketoester **11** with diethyl azodicarboxylate (DEAD) by Cu(II)-(*R,R*)-**1** complex.

Schneider et al. were also concerned with this reaction, for which they used diethyl azodicarboxylate (DEAD) as an amination agent and a chiral copper complex derived from Cu(II) triflate and Bolm’s ligand (*R,R*)-**1** as a catalyst [56]. The reaction of cyclic ester **11** under optimized conditions gave product **48** in 91% yield with 97% ee (Scheme 7).

### 2.8. C-H Functionalization of Indole

**Scheme 8:**
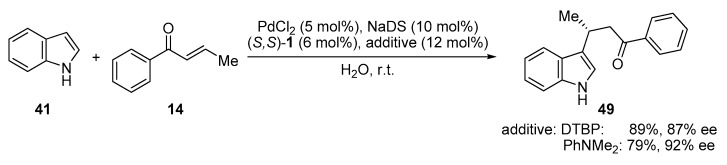
C–H functionalization of indole **42** catalyzed by a Pd(II)-(*S,S*)-**1** complex.

The only application of Bolm’s ligand **1** in the C–H functionalization of indoles was published by Kobayashi et al. in 2016 [57]. The activation of the C3–H bond of indoles was performed by Pd(II) chloride, followed by the reaction with α,β-unsaturated ketones. The reaction was carried out in water with the additive NaDS in order to stabilize the Pd(II)-indolyl intermediate, in order to increase the attractiveness of the described process from the point of view of “green chemistry”. In addition, they found that the presence of water is essential for the fruitful course of the reaction, as it did not give any product in a different solvent. They also screened various organic bases and proton scavengers to prevent the undesired Brønsted acid-mediated pathway [58]. The use of either 2,6-di-tert-butylpyridine (DTBP) or *N,N*-dimethylaniline turned out to be optimal in terms of yield and enantioselectivity. Starting from unsubstituted indole **41** and ketone **14**, the product **49** was obtained in high yields and enantiopurities 87% and 92% ee, respectively (Scheme 8). 

### 2.9. Diels–Alder Reaction

**Scheme 9:**
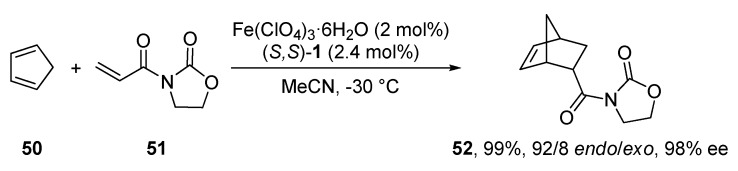
Diels–Alder reaction catalyzed by a Fe(III)-(*S,S*)-**1** complex.

Most recently, Ollevier et al. reported the utilization of Bolm’s ligand **1** in a Lewis acid-catalyzed Diels–Alder reaction [59]. In contrast to the already mentioned Mukaiyama aldol reaction, opening of epoxides, or Michael addition, where Fe(II) salts were compulsory for the activation of the substrate, Fe(III) perchlorate is optimal for high yields and selectivity of the desired products in the case of the Diels–Alder reaction. The reaction gave **52**, the product of cyclization of cyclopentadiene **50** and α,β-unsaturated oxazolidin-2-one **51**, in quantitative yields with high 92/8 *endo*/*exo* ratio and an excellent enantioselectivity of 98% ee using only 2 mol% of the Fe(III)-(*S,S*)-**1** catalyst (Scheme 9).

### 2.10. Other Applications

It should be noted that Bolm’s ligand **1** was also used in a cyclopropanation reaction; however, it did not give satisfactory results, and so another ligand was used for further studies of this reaction [60].

## 3. Conclusions

As an examination of the known literature illustrates, Bolm’s ligand possesses considerable potential to introduce chirality into prochiral substrates in various reactions. Furthermore, progress in enantioselective catalytic procedures using its combination with various metal salts still steadily continues, and it is reasonable to expect further applications. As far as its preparation is concerned, it can could be prepared with a high enantiopurity in just four steps. Hence, it is rather easily available to the chemical community for further studies. 

## Figures and Tables

**Table 1:**
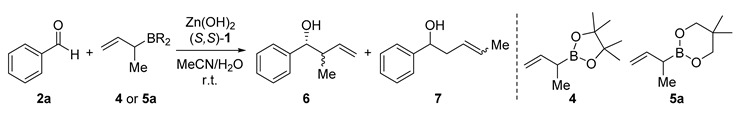
Allylation of benzaldehyde **2a**.

Entry	4 or 5a	Zn(OH)_2_ (mol%)	(*S,S*)-1 (mol%)	MeCN/H_2_O	Yield (%)	6/7	dr (*syn/anti*)	ee (*syn*) (%)
1	**4**	-	-	4/1	77	5/95	-	-
2	**5a**	10	-	4/1	86	45/55	70/30	-
3	**5a**	10	12	7/3	92	>99/1	91/9	71

**Table 2:**
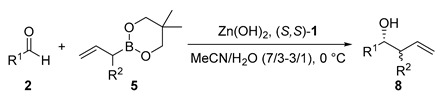
Allylations of benzaldehydes **2** to **8**.

Entry	R^1^		R^2^		Zn(OH)_2_ (mol%)	(*S,S*)-1 (mol%)	Yield (%)	dr (*syn/anti*)	ee (*syn*) (%)
1	PhCH_2_CH_2_	**2b**	Me	**5a**	5	6	94	83/17	85
2	PhCH_2_CH_2_	**2b**	Et	**5b**	5	6	96	75/25	91
3	PhCH_2_CH_2_	**2b**	*i*Bu	**5c**	5	6	95	75/25	91
4	PhCH_2_CH_2_	**2b**	Cl	**5d**	3	3.6	92	93/7	95
5	Ph	**2a**	Cl	**5d**	5	6	92	96/4	88
6	4-MeC_6_H_4_	**2c**	Cl	**5d**	3	3.6	91	96/4	87
7	4-BrC_6_H_4_	**2d**	Cl	**5d**	7.5	9	quant	95/5	85
8	1-naphthyl	**2e**	Cl	**5d**	5	6	94	93/7	85
9	CH_3_(CH_2_)_10_	**2f**	Cl	**5d**	2	2.4	92	93/7	93

**Table 3:**
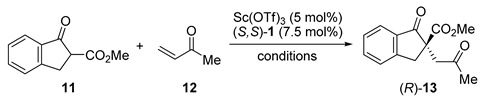
Michael addition of β-ketoester **11** to methyl vinyl ketone (MVK) **12**.

Entry	Solvent ^1^	*T* (°C)	*c* (M)	Yield (%)	ee (%)
1	DCM	10	0.08	22	53
2	DCM	20	0.08	61	80
3	DCM	30	0.08	98	81
4	PhMe	10	0.08	quant.	5
5	MeCN/DCM	30	0.08	76	8
6	DCE	30	0.08	94	84
7	DCE	30	0.04	96	89
8	DCE	40	0.02	94	92

^1^ DCM – dichloromethane, DCE – 1,2-dichloroethane.

**Table 4:**
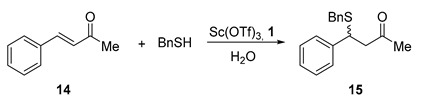
Conjugate addition of benzylthiol on α,β-unsaturated ketone **14**.

Entry	Sc(OTf)_3_ (mol%)	1 (mol%)	Base (mol%)	*T* (°C)	Yield (%)	ee (%)
1	2	5 (*R,R*)	-	30	46	57 (*R*)
2	1	2 (*R,R*)	NaOH (3)	30	95	91 (*R*)
3	1	1.2 (*S,S*)	Pyridine (10)	r.t.	92	93 (*S*)

**Table 5:**
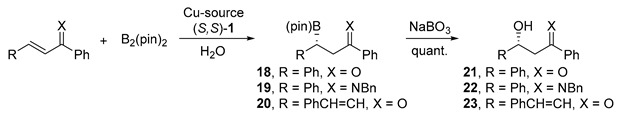
Conjugate boron addition to α,β-unsaturated compounds.

Entry	R	X	Cu-Source	(mol%)	(*S,S*)-1 (mol%)	Additive (mol%)	*T* (°C)	Yield (%)	ee (%)
1	Ph	O	Cu(OH)_2_	5	6	AcOH (6)	5	95	99
2	Ph	O	Cu(OAc)_2_	5	6	MeOH (100)	r.t.	92	92
3	Ph	O	Cu powder	10	12	-	30	92	83
4	Ph	NBn	Cu(OAc)_2_	5	6	-	r.t.	91	>99
5	PhCH=CH	O	Cu(OAc)_2_	5	6	-	5	91	91

**Table 6:**
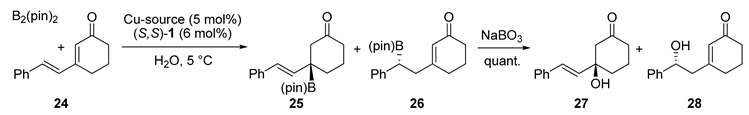
Conjugate addition of B_2_(pin)_2_ to cyclic dienone **24**.

Entry	Cu-Source	Additive (mol%)	Yield (%)	27/28	ee (%)
1	Cu(OH)_2_	-	81	<1/99	76
2	Cu(OH)_2_	AcOH (6 mol%)	92	>99/1	87
3	Cu(OAc)_2_	-	94	>99/1	91

**Table 7:**
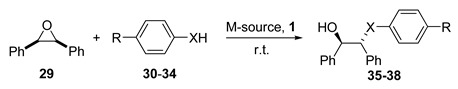
Opening of epoxide ring by amine, alcohol, thiol, and selenol.

Entry	X	R	30–34	(eq.)	M-source ^1^	(mol%)	1 (mol%)	Additive (mol%)	Solvent	Yield (%)	ee (%)
1	NH	H	**30**	1	Sc(OTf)_3_	10	10 (*R,R*)	-	DCM	95	93 (*R,R*)
2	NH	H	**30**	1	Sc(DS)_3_	1	1.2 (*S,S*)	-	H_2_O	89	91 (*S,S*)
3	NH	H	**30**	1.5	Sc(OTf)_3_	10	12 (*S,S*)	-	DCM	85	93 (*S,S*)
4	NH	H	**30**	1.5	Sc(UDST)_3_	10	12 (*S,S*)	-	H_2_O	87	95 (*S,S*)
5	NH	H	**30**	1.5	Cu(OTf)_2_	10	12 (S,S)	-	DCM	18	80 (*R,R*)
6	NH	H	**30**	1.5	Cu(UDST)_2_	10	12 (*S,S*)	-	H_2_O	82	80 (*R,R*)
7	NH	H	**30**	1.5	Zn(OTf)_2_	10	12 (*S,S*)	-	DCM	60	90 (*R,R*)
8	NH	H	**30**	1.5	Zn(UDST)_2_	10	12 (*S,S*)	-	H_2_O	97	92 (*R,R*)
9	NH	H	**30**	1	Zn(OTf)_2_	5	5 (*R,R*)	NaDS (5 mol%)	H_2_O	97	90 (*S,S*)
10	NH	H	**30**	1	ZrCl_4_	5	10 (*R,R*)	NaDS (20 mol%)	H_2_O	55	22 (*R,R*)
11	NH	H	**30**	2	In(OTf)_3_	10	10 (*R,R*)	-	DCM	69	89 (*R,R*)
12	NH	H	**30**	1	Fe(ClO_4_)_2_·6H_2_O	5	6 (*S,S*)	-	DCM	90	95 (*S,S*)
13	CH_2_O	MeO	**31**	2	Sc(OTf)_3_	10	10 (*R,R*)	-	DCM	82	97 (*R,R*)
14	CH_2_O	Br	**32**	1	Sc(DS)_3_	10	12 (*S,S*)	-	H_2_O	34	86 (*S,S*)
15	S	H	**33**	3	Sc(OTf)_3_	10	12 (*S,S*)	-	DCM	84	94 (*S,S*)
16	S	H	**33**	3	Sc(DS)_3_	10	12 (*S,S*)	-	H_2_O	73	89 (*S,S*)
17	S	H	**33**	1.5	InBr_3_	10	11 (*R,R*)	-	DCM	81	96 (*R,R*)
18	Se	H	**34**	3	Sc(OTf)_3_	10	10 (*R,R*)	-	DCM	77	93 (*R,R*)

^1^ DS – dodecyl sulfate, UDST – undecyl sulfonate.

**Table 8:**
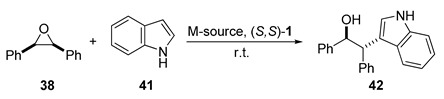
Opening of epoxide ring by indole.

Entry	42 (eq.)	M-source (mol%)		(*S,S*)-1 (mol%)	Solvent	Yield (%)	ee (%)
1	1.2	Sc(OTf)_3_	10	12	DCM	Traces	-
2	1.1	Sc(DS)_3_	5	6	H_2_O	85	93 (*R,R*)
3	1.2	Sc(UDST)_3_	10	12	H_2_O	69	92 (*R,R*)
4	1.2	Cu(OTf)_2_	10	12	DCM	60	86 (*S,S*)
5	1.2	Cu(UDST)_2_	10	12	H_2_O	80	96 (*S,S*)
6	1.2	Zn(OTf)_2_	10	12	DCM	Traces	-
7	1.2	Zn(UDST)_2_	10	12	H_2_O	8	80 (*S,S*)
8 ^1^	1.2	Fe(ClO_4_)_2_·6H_2_O	5	6	DCM	90	>99 (*R,R*)

^1^ 4Å MS were used as an additive.

**Table 9:**
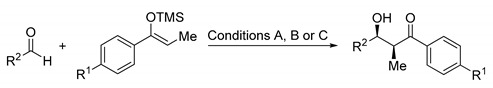
Optimized reaction conditions in Mukaiyama aldol reaction of silyl enol ether with aldehydes.

Silyl Enol Ether		Aldehyde	
	R^2^ = Bulky		R^2^ = Non-Bulky
R^1^	Electron Rich	Electron Poor	
Neutral	A	B	C
EDG	A	C	C
EWG	A	B	C

Conditions A: Fe(OTf)_2_ (3 mol%), (S,S)-**1** (3.6 mol%), pyridine (7.2 mol%), DME/H2O 9/1, 0 °C. Conditions B: Fe(ClO_4_)_2_ (3 mol%), (S,S)-**1** (3.6 mol%), PhCO_2_H (3.6 mol%), DME/H2O 7/3, 0 °C. Conditions C: Bi(OTf)_3_ (3 mol%), (*S,S*)-**1** (9 mol%), pyridine (12 mol%), DME/H_2_O 9/1, 0 °C.

**Table 10:**
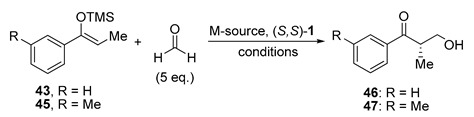
Hydroxymethylation of silyl enol ether **43** and **45** by formaldehyde..

Entry	R	M-source ^1^	(mol%)	(*S,S*)-1 (mol%)	Additive	Solvent	*T* (°C)	Yield (%)	ee (%)
1	H	Sc(OTf)_3_	10	12	-	DCM	−20	80	90
2	H	Bi(OTf)_3_	1	3	2,2′-bipyridine (5 mol%)	H_2_O	0	93	91
3	Me	Sc(DS)_3_	10	12	Triton^®^ X-705	H_2_O	r.t.	73	90

^1^ DS – dodecyl sulfate.
